# IL-9 and Blimp-1 protects the transcriptional identity of group 2 innate lymphocytes in allergic asthma

**DOI:** 10.21203/rs.3.rs-6365283/v1

**Published:** 2025-04-11

**Authors:** Yibo Zheng, Jinyi Zhang, Aaron Yang, Kun He, Arianna Creech, Daniella M. Schwartz, Rachel A. Gottschalk, Nicolas Bouladoux, Yasmine Belkaid, Amanda C. Poholek

**Affiliations:** 1 Center for Systems Immunology, Department of Immunology, University of Pittsburgh School of Medicine, Pittsburgh, PA; 2 Tsinghua University School of Medicine, Beijing, China; 3 Program in Microbiology and Immunology, University of Pittsburgh School of Medicine, Pittsburgh, PA; 4 Metaorganism Immunity Section, Laboratory of Host Immunity and Microbiome, National Institute of Allergy and Infectious Diseases, National Institutes of Health, Bethesda, MD; 5 Metaorganism Unit, Immunology Department, Institut Pasteur, Paris, France; 6 Division of Rheumatology, Department of Medicine, University of Pittsburgh School of Medicine, Pittsburgh, PA; 7 Lead Contact

## Abstract

Allergic asthma is driven by type 2 immune cells including type 2 innate lymphoid cells (ILC2s). ILC2s respond to the tissue alarmins IL-33 and IL-25, however these signals do not uniquely promote type 2 inflammation, and the factors that maintain ILC2s ability to produce type 2 cytokines are not known. Here, we show that allergen-driven tissue alarmins IL-33 and IL-25 rapidly induce IL-9, which directly upregulates the transcriptional repressor Blimp-1 through an autocrine/paracrine mechanism. Blimp-1 promotes type 2 responses by directly repressing type 1 inflammation including the cytokines IFNγ and TNF. Deletion of Blimp-1 in ILC2s increases type 1 cytokines and concomitantly reduces type 2 cytokines, ameliorating mucus production and airway inflammation in response to allergens. Thus, Blimp-1 maintains the type 2 transcriptional identity of ILC2s in response to inflammation, driving type 2 immunity and allergic asthma.

Allergic asthma is a chronic inflammatory disease of the lungs that affects millions of adults and children worldwide^1^. It is characterized by bronchoconstriction, mucus plugging of the airways, smooth muscle remodeling, reversible airflow obstruction, and easily triggered bronchospasms^2^. Type 2 cytokines such as IL-5, IL-9 and IL-13 play critical roles in promoting the inflammatory response that underlies the pathophysiologic consequences of allergic disease. Both Th2 cells and group 2 innate lymphoid cells (ILC2s) produce type 2 cytokines and have been implicated as drivers in the pathogenesis of asthma. ILC2s respond to tissue damage signals locally in the lung tissue, and through the secretion of type 2 cytokines enhance Th2 responses and amplify their effects on lung tissue^2-5^. However, tissue alarmins do not uniquely promote type 2 inflammatory responses, and inflammatory states associated with type 1 responses can shift ILC2s towards an ILC1 state^6-8^.

B lymphocyte–induced maturation protein-1 (Blimp-1) is a transcriptional repressor with wide ranging roles in immune cells. Initially identified as the master regulator of plasma cells^9^, Blimp-1 also plays significant roles in shaping CD8 T cell effector, exhaustion and memory responses, as well as CD4 T cells and NK cells, where it helps regulate effector differentiation and function^10,11^. We recently reported an unexpected role for Blimp-1 in the context of inhaled allergens where Blimp-1 was required for the development of Th2 cells that migrate to the lung and cause allergic asthma^12,13^. Given the shared features between Th2 cells and ILC2s in the context of allergic asthma, we hypothesized that Blimp-1 may also play a role within the ILC2s in response to allergens. While Blimp-1 expression in a subset of IL-10 producing ILC2s has been reported^14^, the specific role of Blimp-1 in ILC2s has not been thoroughly investigated.

In this study, we identify a role for Blimp-1 in ILC2s to promote allergic asthma by maintaining their type 2 inflammatory identity during tissue inflammation. Tissue damage signals IL-33 and IL-25 promote expression of IL-9 which is necessary and sufficient to induce Blimp-1. ILC2 cells deficient in Blimp-1 exhibited an increase in TNF, IFNγ and other type 1 inflammatory cytokines and chemokines leading to a decrease in type 2 inflammation and pathophysiologic features of allergic asthma. Our study suggests that in response to tissue inflammation, ILC2s require transcriptional repression of alternate fates to limit plasticity. Finally, this study adds ILC2s to the cell types in which the transcriptional repressor Blimp-1 plays important roles in effector differentiation, illuminating a pathway that offers potential therapeutic targets for asthma treatment.

## Results:

### Blimp-1 is expressed in ILC2s during lung inflammation.

**Extended Data Fig. 1:**
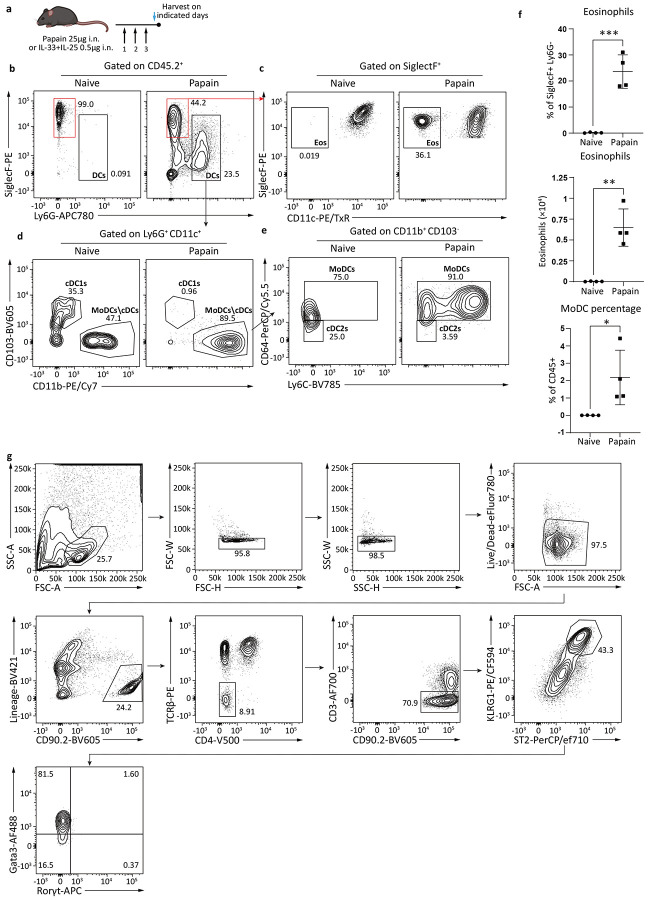
Papain drives lung inflammation and activates ILC2s to express Blimp-1. **a**, Schematic for papain induced asthma model. **b-e**, Gating strategy for eosinophils (**c**), cDC1s (**d**), cDC2s and MoDCs (**e**). **f**, Quantification of eosinophil percentage, eosinophil number and MoDC number in BAL after papain. **g**, Gating strategy for ILC2s. Each point represents one individual sample. The data are shown as means ± s.d., and present two or three independent experiments. A two-tailed unpaired t test was performed for **f**. *P < 0.05, **P < 0.01, ***P < 0.001. The specific P values are as follows for **f**: comparison between eosinophil percentage, P = 0.0003; comparison between total eosinophils, P = 0.0012; for E, P = 0.0323.

**Fig. 1:**
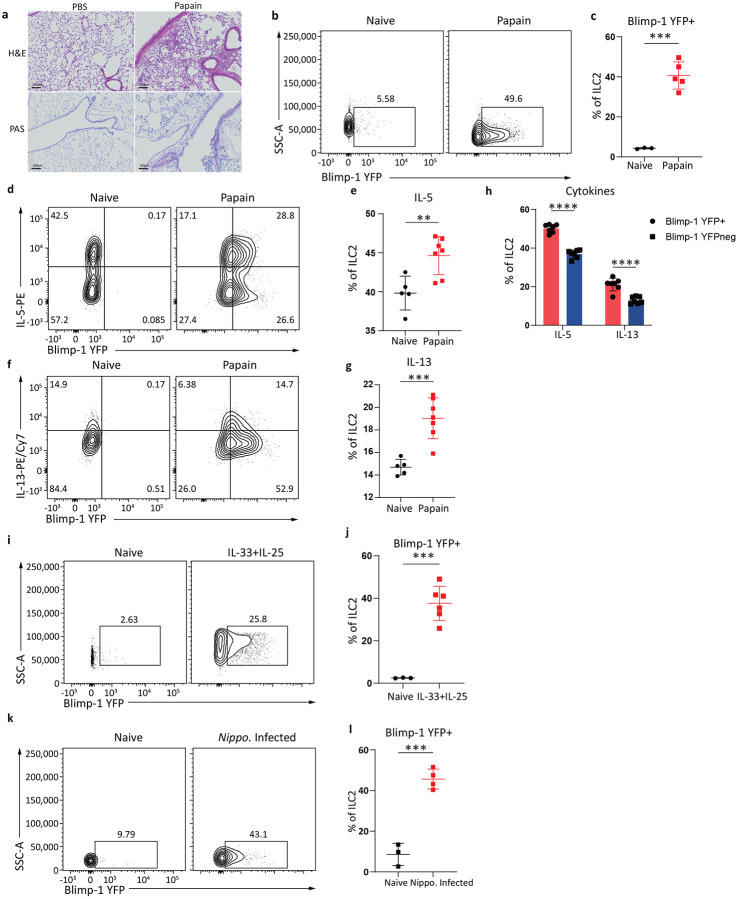
Blimp-1 is expressed in activated lung ILC2s in response to tissue inflammation and infection. **a**, Representative H&E and PAS staining of lungs from indicated groups. Lung samples were fixed for histology (n=5) **b**, Representative flow plot of Blimp-1-YFP in ILC2s **c**, Quantification of (**b**). **d** and **f**, Representative flow plots of Blimp-1 YFP and IL-5 (**d**) or IL-13 (**f**) **e** and **g**, Quantification of IL-5+ (**e**) or IL-13+ (**g**) ILC2 from (**d** and **f**). **h,** Percentage of IL-5 and IL-13 expression in Blimp-1 YFP+ ILC2 or Blimp-1 YFP- ILC2. **i** and **k**, Representative flow plots of Blimp-1 YFP in ILC2s isolated from indicated groups. **j** and **l**, Quantification of Blimp-1 YFP+ ILC2s from (**i**, **k**). Each point represents one individual sample. The data are shown as means ± s.d., and present two or three independent experiments. A two-tailed unpaired t test was performed for **b**, **e**, **g**, **j** and **l**. Šídák's multiple comparisons test was performed for **h**. *P < 0.05, *P < 0.01, ***P < 0.001, ****P < 0.0001. The specific P values are as follows: for **b**: P = 0.0001; for **e**: P = 0.0055; for **g**: P = 0.0005; for **h**: comparison between IL-5, P < 0.0001, comparison between IL-13, P < 0.0001; for **j**: P = 0.0002; for **l**: P = 0.0002.

We previously showed Blimp-1 is required for development of Th2 cells in response to inhaled allergens in a murine model of house dust mite induced allergic asthma^12,13^. Given the importance of ILC2s in allergic lung inflammation, we considered that Blimp-1 may also play a role in ILC2 biology driving lung disease. Using Blimp-1 YFP reporter animals, we measured the expression of Blimp-1 in ILC2s using a papain driven model of allergic asthma (Extended Data Fig.1a). Papain is a cystine protease that robustly activates ILC2s and is considered a non-T cell driven model of allergic asthma^15-17^. Three days of consecutive intranasal (i.n.) papain administration resulted in lung inflammation, mucus in the airways, and inflammatory cellular infiltrate measured by eosinophil and dendritic cell recruitment (Fig. 1a, Extended Data Fig.1b-f) as expected. Blimp-1 was robustly and significantly expressed after papain treatment as measured by YFP expression in ST2^+^ KLRG1^+^ GATA3^+^ ILC2 cells. (Fig. 1b-c, Extended Data Fig.1g). Papain activated ILC2s leading to increased type 2 cytokine expression and Blimp-1+ ILC2s expressed both IL-5 and IL-13 (Fig. 1d-g). Though not all Blimp-1+ ILC2s expressed cytokines, Blimp-1+ cells produced significantly more IL-5 and IL-13 than Blimp-1 negative cells (Fig. 1h), suggesting Blimp-1 may be expressed in the most differentiated effector ILC2s, in line with Blimp-1’s function in T and B cell lineages^18^.

IL-33 and IL-25 are alarmins produced in response to tissue damage that potently activate ILC2s and i.n. administration promotes lung inflammation^19^. To determine if IL-33 and IL-25 could induce Blimp-1 in ILC2s in vivo, we administered recombinant IL-33 and IL-25 i.n. for three days similar to the papain model. Indeed, i.n. IL-33 and IL-25 robustly induced Blimp-1 in ILC2s (Fig. 1i-j). ILC2s are also critical for worm clearance^20,21^. To test if Blimp-1 is expressed in ILC2s responding to worm infection, we used *Nippostrongylus brasilensis* as a model that includes infection of both the lung and gastrointestinal tract^22^. Similar to i.n. papain and IL-33+IL-25 activation of ILC2s, Blimp-1 was expressed in lung ILC2s five days after infection (Fig. 1k-l). These data suggested that Blimp-1 is expressed in effector ILC2s in response to allergens, tissue alarmins, and worm infection, suggesting Blimp-1 may play a previously unappreciated role in ILC2 biology.

### Blimp-1 is expressed in ILC2s across barrier and mucosal tissues.

**Extended Data Fig. 2:**
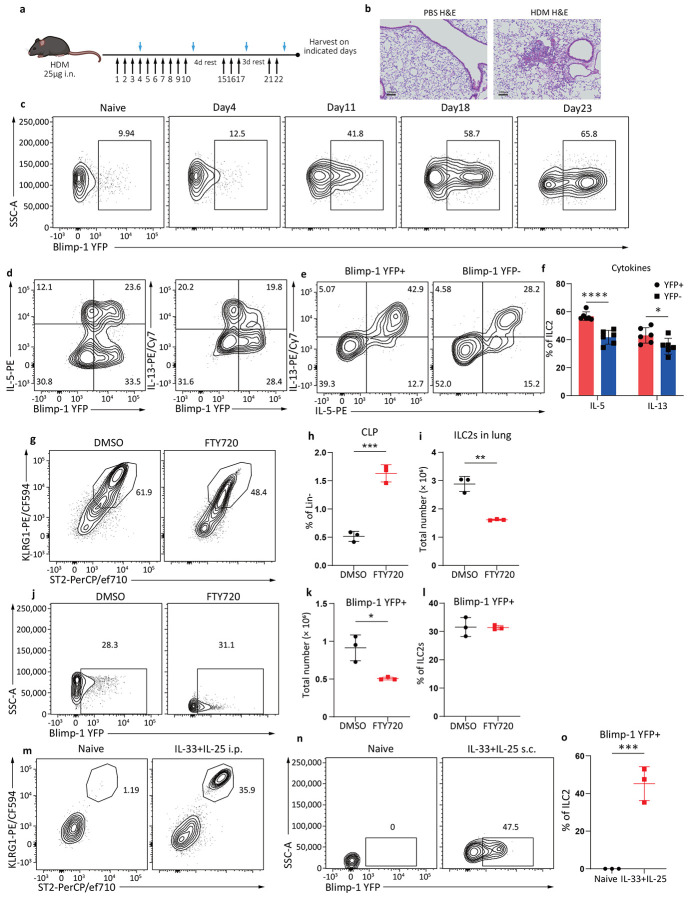
HDM drives lung inflammation and activates ILC2s to express Blimp-1 with a time dependent manner. **a**, Schematic for HDM driven asthma model. **b**, Representative H&E staining after HDM. **c**, Percentage of Blimp-1 YFP in ILC2s at indicated timepoints by HDM model. **d**-**e**, Blimp-1 co-expression with IL-5 or IL-13 (**d**), or IL-5 and IL-13 co-expression (**e**) after HDM. **f,** Quantification of (e). **g** and **j**, Percentage of ILC2s in the lung (**g**) and Blimp-1 YFP in ILC2s (**j**) after FTY720 treatment. **h**-**i** and **k**-**l**, Quantification of CLP in bone marrow (**h**), ILC2s number in lung (**i**), Blimp-1 YFP+ ILC2s number (**k**) and percentage of Blimp-1 YFP+ ILC2s (**l**). **m**, Percentage of ILC2s from mesenteric lymph nodes with IL-33&25 i.p.. **n**, Percentage of Blimp-1 YFP in ILC2s from skin draining lymph nodes after IL-33&25 s.c..**o**, Quantification of (**n**). Each point represents one individual sample. The data are shown as means ± s.d., and present two or three independent experiments. A two-tailed unpaired t test was performed for **h**, **i**, **k**, **l** and **o**. Šídák's multiple comparisons test was performed for **f**. *P < 0.05, **P < 0.01, ***P < 0.001, ****P < 0.0001. The specific P values are as follows: for **f**, comparison between IL-5, P < 0.0001, comparison between IL-13, P = 0.0255; for **h**, P = 0.0004; for **i**, P = 0.0012; for **k**, P = 0.0149; for **l**, P = 0.9245; for **o**, P = 0.001.

**Fig. 2:**
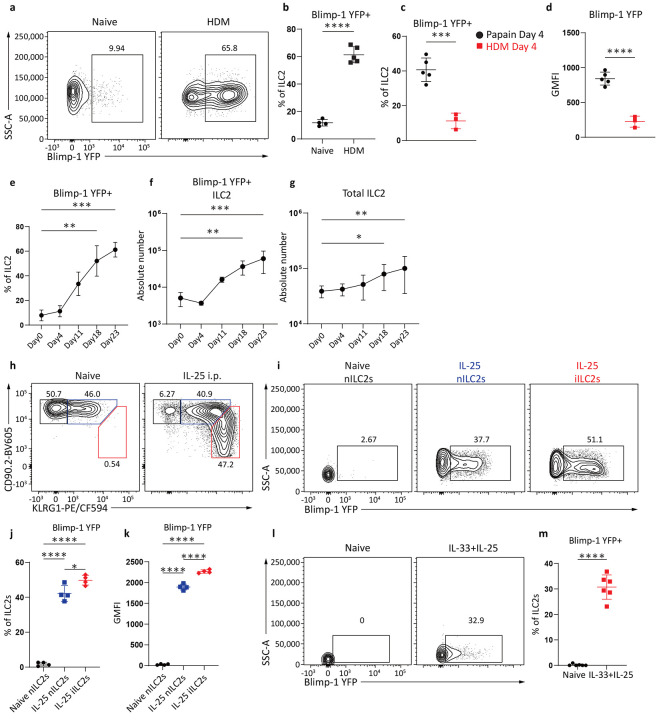
Blimp-1 is expressed in ILC2s across barrier and mucosal tissues. **a**, Percentage of Blimp-1 YFP in ILC2s after HDM. **b**, Quantification of (**a**) (n=5) **c**-**d**, Quantification of Blimp-1 YFP percentage (**c**) and GMFI (**d**) after HDM or papain on day 4. **e**-**g**, Percentage (**e**), absolute number (**f**) of Blimp-1 YFP in ILC2s, or absolute number of ILC2s (**g**) at indicated timepoints. **h**, Representative flow plot for iILC2 gating after IL-25 i.p.. (n=4) **i**, Percentage of Blimp-1 YFP in ILC2 subsets from (**h**) **j**-**k,** Quantification of Blimp-1 YFP percentage (**j**) or GMFI (**k**) in nILC2 and iILC2. **l,** Percentage of Blimp-1 YFP in gut ILC2 after IL-33 and IL-25 i.p.. (n=6) **m,** Quantification of(**l**). Each point represents one individual sample. The data are shown as means ± s.d., and present two or three independent experiments. A two-tailed unpaired t test was performed for**b**, **c**, **d** and **m**. Tukey’s multiple comparisons test was performed for **j**, and**k**, and Kruskal-Wallis multiple comparisons test was performed for **e**, **f** and **g**. *P < 0.05, *P < 0.01, ***P < 0.001, ****P < 0.0001. The specific P values are as follows for **b**: P < 0.0001; for **c**: P = 0.0006; for **d**: P < 0.0001; for **e**, day0 vs. day18, P = 0.0016, day0 vs. day23, P = 0.0001; for **f**, day0 vs. day18, P = 0.0054, day0 vs. day23, P = 0.001; for **g**, day0 vs. day18, P = 0.0385, day0 vs. day23, P = 0.0084; for **j**: IL-25 iILC2s vs. IL-25 nILC2s, P = 0.0184, IL-25 iILC2s vs. Naive nILC2s, P < 0.0001, IL-25 nILC2s vs. Naive nILC2s, P < 0.0001; for **k**: IL-25 iILC2s vs. IL-25 nILC2s, P < 0.0001, IL-25 iILC2s vs. Naive nILC2s, P < 0.0001, IL-25 nILC2s vs. Naive nILC2s, P < 0.0001; for **m**: P < 0.0001.

House dust mite (HDM) is a common allergen that we previously showed induced expression of Blimp-1 in Th2 cells^23^. Using a chronic inhaled HDM model that drives robust Th2-driven lung inflammation (Extended Data Fig.2a-b), we found HDM also significantly induced Blimp-1 in ILC2s (Fig. 2a-b). Surprisingly, HDM induced Blimp-1 to even a larger extent than papain stimulation, as nearly 65% of ILC2s in the lung expressed Blimp-1 after two rounds of HDM rechallenge (Extended Data Fig.2c). Similar to papain, Blimp-1+ ILC2s expressed more IL-5 and IL-13 than Blimp-1 negative ILC2s, though not all Blimp-1+ cells expressed cytokines (Extended Data Fig.2d-f). We wondered if this increase was related to the cumulative effect of chronic administration of HDM despite the fact that ILC2s are rapidly activated and secrete cytokines in response to alarmins. Indeed, less Blimp-1 was expressed after three doses of HDM (day 4) compared to three doses of papain (Fig. 2c-d). Rechallenges at day 18 and 23 further increased Blimp-1 expression both in percentage and number, while the total number of ILC2s only moderately increased (Fig. 2e-g). These data suggested either cumulative signals promoted additional Blimp-1 expression within the tissue residing ILC2 pool or the recruitment of new Blimp-1+ ILC2s into the lung.

Inflammatory ILC2s (iILC2s; CD90.2^lo^ KLRG1^hi^) are a recently described population of ILC2s recruited to the lung from the gastrointestinal (GI) tract in response to inflammatory signals and are distinct from natural ILC2 cells (nILC2s; CD90.2^hi^ KLRG1^lo^) that reside in the lung^24,25^. ILC2s are limited in naïve tissues but rapidly appear at several sites in response to administration of alarmins such as IL-25 or *Nippostrongylus brasilensis* infection^26 27^. We wondered if Blimp-1 expression in ILC2s was specific to either the iILC2s or nILC2s in the lung and may account for the different expression profiles between HDM and papain allergic asthma models. iILC2s were readily found in the lung after IL-25 administration (Fig. 2h). Blimp-1 was upregulated in both iILC2s and nILC2s, however iILC2s expressed slightly more Blimp-1 than nILC2s (Fig. 2i-k).

ILC2s are also recruited from the bone marrow and can be blocked with FTY720, an agonist of S1P1^28,29^. Blocking ILC2 recruitment with FTY720 reduced the total number of ILC2s in the lung, while precursors were increased in the bone marrow after IL-33 treatment (Extended Data Fig.2g-i). Interestingly, blocking recruitment of ILC2s did not affect the percentage of Blimp-1 positive cells in the lung, despite their reduced number (Extended Data Fig.2j-l). Taken together, these data suggest that Blimp-1 expression is increased in ILC2s locally proliferating in the lung tissue, as well as in those ILC2s that are recruited into the lung from other sites such as the GI tract and the bone marrow, likely accounting for cumulative increases in Blimp-1+ ILC2s over time in response to HDM.

We next confirmed if Blimp-1 expression occurred in ILC2s in other tissue sites. To activate ILC2 cells in the small intestinal lamina propria (siLP), we treated animals i.p. with IL-33 and IL-25. After 3 days of stimulation, ILC2s were found in the mesenteric lymph nodes that expressed Blimp-1 (Fig. 2l-m, Extended Data Fig.2m). Skin, as a barrier organ, also contains ILC2^30-32^. Subcutaneous injection of IL-33 and IL-25 expanded the ILC2 population in the draining lymph nodes, which also drove expression of Blimp-1 (Extended Data Fig.2n-o). These data suggest Blimp-1 upregulation in ILC2s is a global feature across barrier and mucosal tissues sites in response to inflammatory tissue alarmins.

### IL-33 and IL-25 indirectly drives Blimp-1 expression in activated ILC2s.

**Extended Data Fig. 3:**
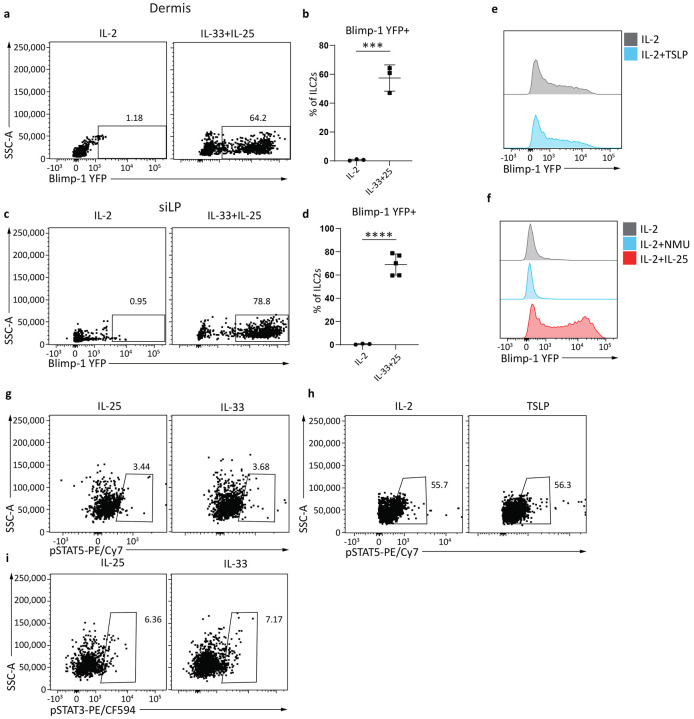
Blimp-1 can be induced in gut and skin ILC2s in vitro. **a**, Percentage of Blimp-1 YFP in dermis ILC2s after IL-33&25. **b**, Quantification of (**a**). **c**, Percentage of Blimp-1 YFP in siLP ILC2s after IL-33&25. **d**, Quantification of (**d**). **e**, Histogram for Blimp-1 YFP in ILC2s after TSLP stimulation. **f**, Histogram for Blimp-1 YFP in ILC2s after NMU or IL-25 stimulation. **g**-**i**, Percentage of phosphorylated STAT5 stimulated with IL-33 (**g**) or TSLP (**h**), or percentage of phosphorylated STAT3 stimulated with IL-33 (**i**). (n=6) Each point represents one individual sorting sample from 2 pooled animals. The data are shown as means ± s.d., and present two or three independent experiments. A two-tailed unpaired t test was performed for **b** and **d**. *P < 0.05, *P < 0.01, ***P < 0.001, ****P < 0.0001. The specific P values are as follows: for **b**, P = 0.0004; for **d**, P < 0.0001.

**Fig. 3:**
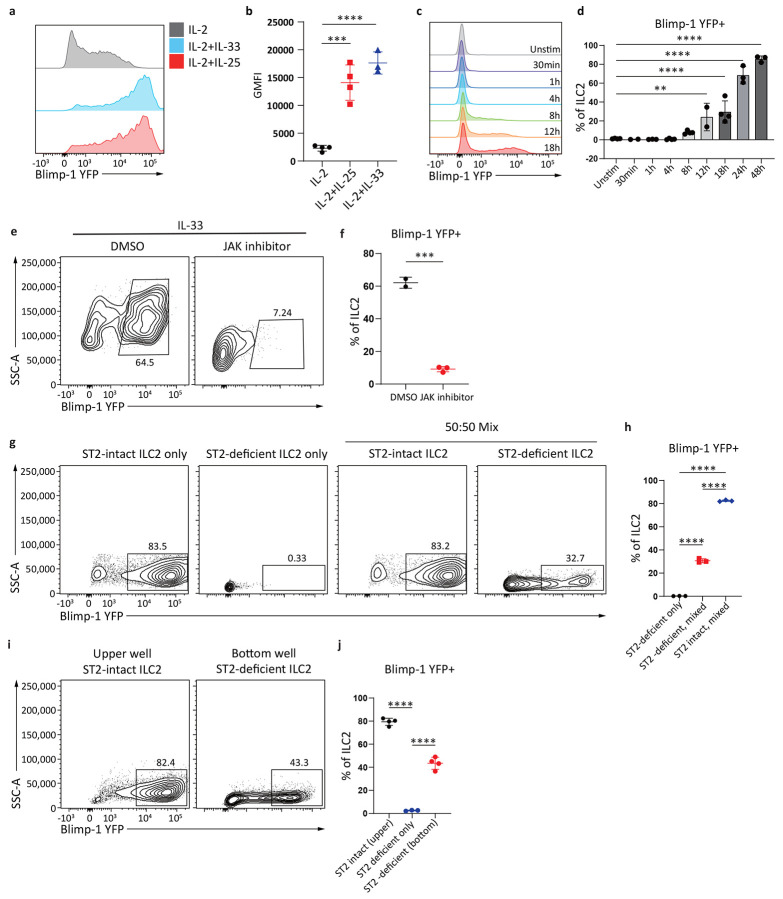
IL-33 and IL-25 indirectly upregulate Blimp-1 in ILC2s. **a**, Histogram for Blimp-1 YFP in ILC2s after stimulated with IL-33 or IL-25 for 3 days. **b**, Quantification of Blimp-1 GMFI from (**a**). (n=4) **c**, Histogram for ILC2s Blimp-1 expression at indicated time points when stimulated with IL-33&25. **d**, Quantification of(**c**). (n=4) **e**, Percentage of Blimp-1 YFP in stimulated ILC2s when treated with JAK inhibitor. **f**, Quantification of (**e**). (n=4) **g**, Percentage of Blimp-1 YFP in ILC2s from ST2 KO&WT ILC2 cell mixing experiment. **h**, Quantification of (**g**). **i**, Percentage of Blimp-1 YFP in ILC2s from ST2 KO&WT ILC2 transwell assay. **j**, Quantification of (**i**). Each point represents one individual sorting sample from 2 pooled animals. The data are shown as means ± s.d., and present two or three independent experiments. A two-tailed unpaired t test was performed for **f**. Dunnett's multiple comparisons test was performed for **b**, **d** and **j**. Tukey’s multiple comparisons test was performed for **h**. *P < 0.05, *P < 0.01, ***P < 0.001, ****P < 0.0001. The specific P values are as follows: for **b**: IL-2 vs. IL-2+IL-25, P = 0.0001, IL-2 vs. IL-2+IL-33, P < 0.0001; for **d**, Unstim vs. 12h, P = 0.0028, Unstim vs. 18h, P < 0.0001, Unstim vs. 24h, P < 0.0001, Unstim vs. 48h, P < 0.0001; for **f**, P = 0.0002; for **h**: ST2 KO ILC2s, mixed vs. ST2 WT ILC2s, mixed, P < 0.0001, ST2 KO ILC2s, mixed vs. ST2 KO ILC2s only, P < 0.0001, ST2 WT ILC2s, mixed vs. ST2 KO ILC2s only, P < 0.0001; for **j**: ST2 KO only vs. Transwell ST2 KO, P < 0.0001, ST2 KO only vs. Transwell ST2 WT, P < 0.0001.

We next sought to understand what factors specifically support the upregulation of Blimp-1 in ILC2s. As IL-33 and IL-25 in vivo drove Blimp-1 in ILC2s, we reasoned that stimulation of ILC2s in vitro with these alarmins may also lead to Blimp-1 upregulation. Sorted lung ILC2 from naive Blimp-1 YFP animals were stimulated in vitro for three days with IL-33 or IL-25 in addition to IL-2 to support ILC2 survival. IL-2 alone increased limited amounts of Blimp-1, however IL-33 or IL-25 potently induced Blimp-1 in nearly all ILC2s (Fig. 3a-b). ILC2s sorted from the dermis and siLP stimulated in vitro with IL-33 also readily expressed Blimp-1 (Extended Data Fig.3a-d). Remarkably, and in contrast to the expression kinetics in vivo in response to HDM, time course analysis demonstrated IL-33 could induce Blimp-1 as early as 8 hours, and more than 50% of ILC2s were Blimp-1+ by 24 hours (Fig. 3c-d). TSLP and NMU are also known activators of ILC2s, however neither one induced Blimp-1 above IL-2 alone (Extended Data Fig.3e-f).

In T cells, Blimp-1 is driven by cytokine-Jak/STAT pathways such as IL-12/STAT4 in Th1 cells, and IL-10/STAT3 in Th2 cells^33,34^. To test if Blimp-1 is also driven by STAT pathways in ILC2 cells, sorted lung ILC2 from naive Blimp-1 YFP animals were stimulated in vitro with IL-33 for 24 hrs with or without a JAK inhibitor. Inhibition of STAT pathways via the JAK inhibitor completely ablated Blimp-1 expression (Fig. 3e-f) suggesting the Jak/STAT pathways are critical for Blimp-1 upregulation. IL-33 and IL-25 have been reported to activate STATs, however we were unable to detect canonical phospho-STAT3 or STAT5 activation downstream of these alarmins in ILC2s (Extended Data Fig.3g-i).

Given the time course of Blimp-1 upregulation, we hypothesized that Blimp-1 may be indirectly regulated by IL-33 and IL-25 by another factor rapidly upregulated in activated ILC2s. To test this, we mixed IL-33 receptor (ST2) deficient ILC2s 1:1 with ST2 intact cells and stimulated them with IL-33. ST2-deficient ILC2s cultured alone were unable to induce Blimp-1 and also failed to become activated, while ST2-intact cells robustly expressed Blimp-1 as expected (Fig. 3g). Remarkably, ST2-deficient ILC2s cultured together with ST2-intact cells produced a significant amount of Blimp-1, though less than ST-intact cells (Fig. 3g-h). To address whether this indirect Blimp-1 upregulation is by direct cell-cell interaction or by secreted factors, we repeated the co-culture experiment using transwells to limit direct contact between ILC2 populations. In the absence of cell-cell interaction, ST2-deficient ILC2s still produced significant amounts of Blimp-1 (Fig. 3i-j). These data suggest IL-33 and IL-25 indirectly drive Blimp-1 expression in ILC2s, likely through upregulation of a secreted factor that can activate STAT family members.

### IL-9 is necessary and sufficient to induce Blimp-1 in ILC2 cells

**Fig. 4:**
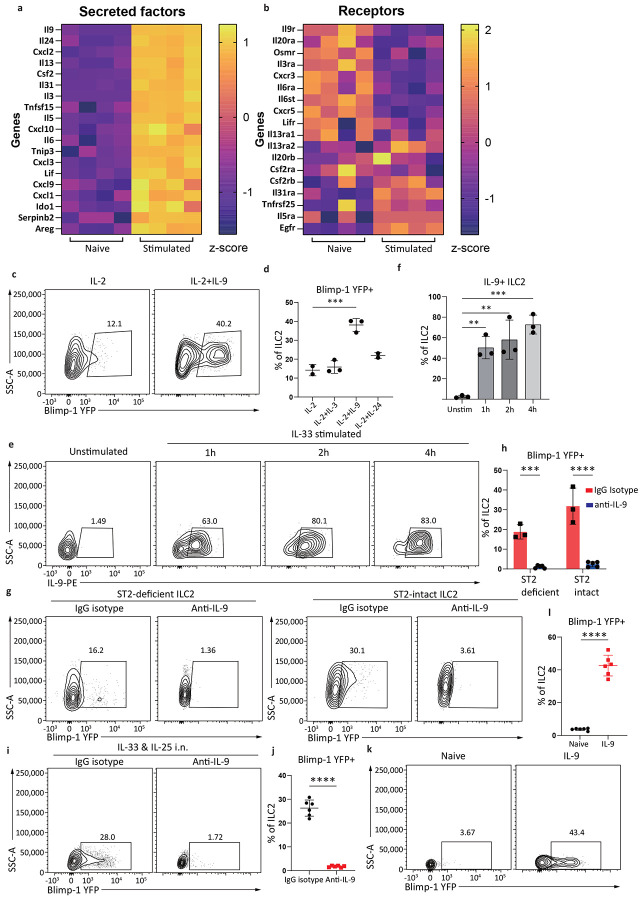
IL-9 is the indispensable driver of Blimp-1 in ILC2s. **a**-**b**, RNAseq comparing naïve ILC2s with ILC2s stimulated for 24 hrs with IL-33. Z-scores were calculated by TPM. **c**, Percentage of Blimp-1 YFP in ILC2s stimulated with IL-3, IL-9 or IL-24 for 24 hours. (n=3) **d**, Quantification of (**c**). **e**, Percentage of IL-9+ ILC2s at indicated timepoints when stimulated with IL-33. (n=3) **f**, Quantification of (**e**). **g**, Percentage of Blimp-1 YFP in ILC2s from transwell assay after treated with anti-IL-9 blocking antibody. **h**, Quantification of (**g**). (n=3) **i**, Percentage of Blimp-1 YFP in ILC2s after anti-IL-9 blockade in vivo. (n=6) **j**, Quantification of (**i**). **k**, Percentage of Blimp-1 YFP in ILC2s after IL-9 treatment for 3 days. (n=6) **l**, Quantification of (**k**). Each point represents one individual sorting sample from 2 pooled animals. The data are shown as means ± s.d., and present three or four independent experiments. A two-tailed unpaired t test was performed for **j** and **l**. Dunnett's multiple comparisons test was performed for **d** and **f**. Šídák's multiple comparisons test was performed for **h**. *P < 0.05, *P < 0.01, ***P < 0.001, ****P < 0.0001. The specific P values are as follows: for **d**, IL-2 vs. IL-2+IL-9, P = 0.0004; for F, Unstimulated vs. 1h, P = 0.003, Unstimulated vs. 2h, P = 0.0012, Unstimulated vs. 4h, P = 0.0002; for **h**, comparison within ST2 KO, P = 0.0001, comparison within ST2 WT, P < 0.0001; for **j**, P < 0.0001; for **l**, P < 0.0001.

To determine candidate secreted factors from ILC2s capable of promoting Blimp-1, we performed bulk RNAseq comparing unstimulated ILC2s ex vivo from lung with ILC2s stimulated with IL-33 for 24 hrs. Genes for secreted proteins significantly upregulated within 24hrs were selected and compared to the expression of their cognate receptors on unstimulated ILC2s (Fig. 4a-b). Based on these data, we selected three top candidate genes for further testing: the cytokines IL-9, IL-24 and IL-3. ILC2s were sorted from Blimp-1 YFP animals and stimulated for 24 hrs with IL-2 alone or IL-2 plus each candidate cytokine. IL-9 drove significantly more Blimp-1 than IL-2 alone or the other candidates, IL-3 and IL-24 (Fig. 4c-d). IL-9 is known to be robustly expressed by ILC2s^35^, however given the timeline of Blimp-1 expression (Fig. 3c) we wondered how quickly IL-9 was produced by ILC2s in response to IL-33. We found ILC2s robustly produced IL-9 within one hour after IL-33, and by 4 hrs nearly 85% of ILC2s expressed IL-9 (Fig. 4e-f).

To investigate whether IL-9 is necessary for Blimp-1 expression, we performed transwell assays as above with ST2-deficient ILC2s in the lower well, and ST-intact ILC2s in the upper well followed by IL-33 stimulation with or without blocking anti-IL-9. IL-9 blockade completed abrogated Blimp-1 expression in both ST2-intact and ST2-deficient ILC2s, demonstrating that IL-9 was essential for Blimp-1 expression in ILC2 cells (Fig. 4g-h). Next, we administered IL-33+IL-25 i.n. with or without IL-9 blockade. Blimp-1 expression in ILC2s in the lung was completed abolished when IL-9 was blocked (Fig. 4i-j). Finally, to determine if IL-9 was sufficient to induce Blimp-1 in vivo, we administered recombinant IL-9 i.n three times similar to IL-33 or papain and found this was sufficient to drive Blimp-1 in ILC2 (Fig. 4k-l). Collectively these data demonstrate that IL-9 is the primary driver of Blimp-1 in ILC2s in response to activation by alarmins.

### Blimp-1 suppresses Type1 genes in ILC2s.

**Extended Data Fig. 4:**
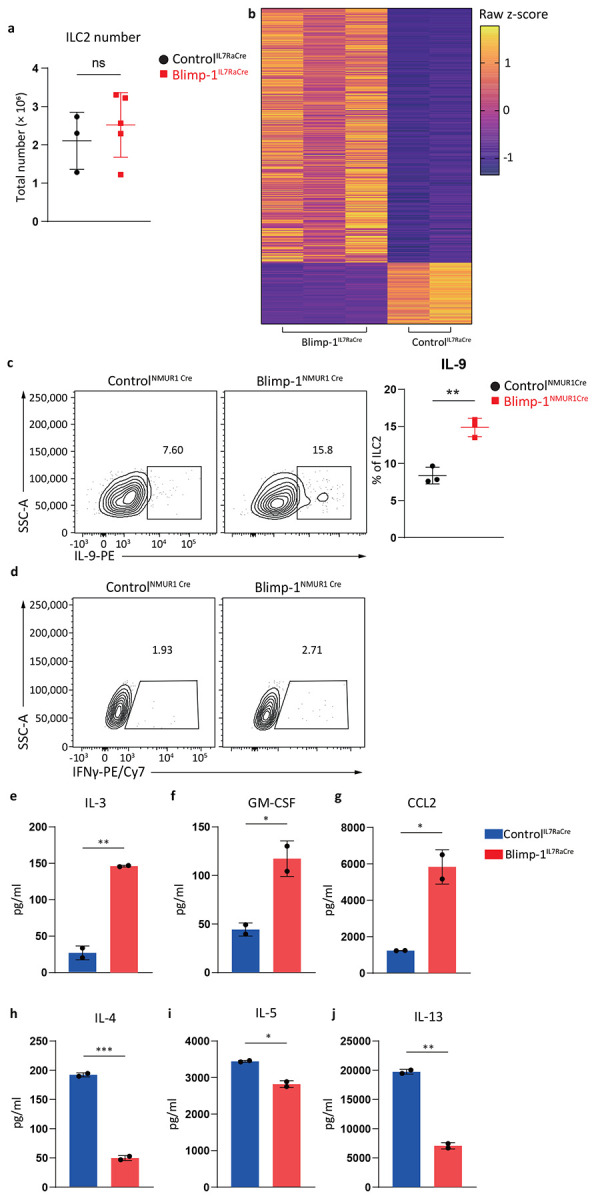
Loss of Blimp-1 in ILC2s in vitro alters cytokines and transcriptomes. **a**, Quantification of Control^IL7RaCre^ or Blimp-1^IL7RaCre^ naïve state ILC2s number. **b**, Heatmap from RNA sequencing comparing Control^IL7RaCre^ with Blimp-1^IL7RaCre^ ILC2s. Z-scores were calculated by TPM. **c**, Percentage of IL-9+ ILC2s from Control^NMUR1Cre^ or Blimp-1^NMUR1Cre^ . (n=3) **d**, Percentage of IFNγ+ ILC2s from Control^NMUR1Cre^ or Blimp-1^NMUR1Cre^. **e**-**j**, Quantification of ILC2s IL-3 (**e**), GM-CSF (**f**), CCL2 (**g**), IL-4 (**h**), IL-5 (**i**) and IL-13 (**j**) production comparing Control^IL7RaCre^ with Blimp-1^IL7RaCre^. (n=2) Each point represents one individual sorting sample from 2 pooled animals. The data are shown as means ± s.d., and present two or three independent experiments. A multiple unpaired t-test was performed for **a**, **c**, **e**, **f**, **g**, **h**, **i** and **j**. *P < 0.05, *P < 0.01, ***P < 0.001, ****P < 0.0001. The specific P values are as follows: for **a**, P = 0.9244; for **c**, P = 0.0025; for **e**, P = 0.0071; for **f**, P = 0.0423; for **g** P = 0.0275; for **h**, P = 0.0038; for **i**, P = 0.0216; for **j**, P = 0.0038.

**Fig. 5:**
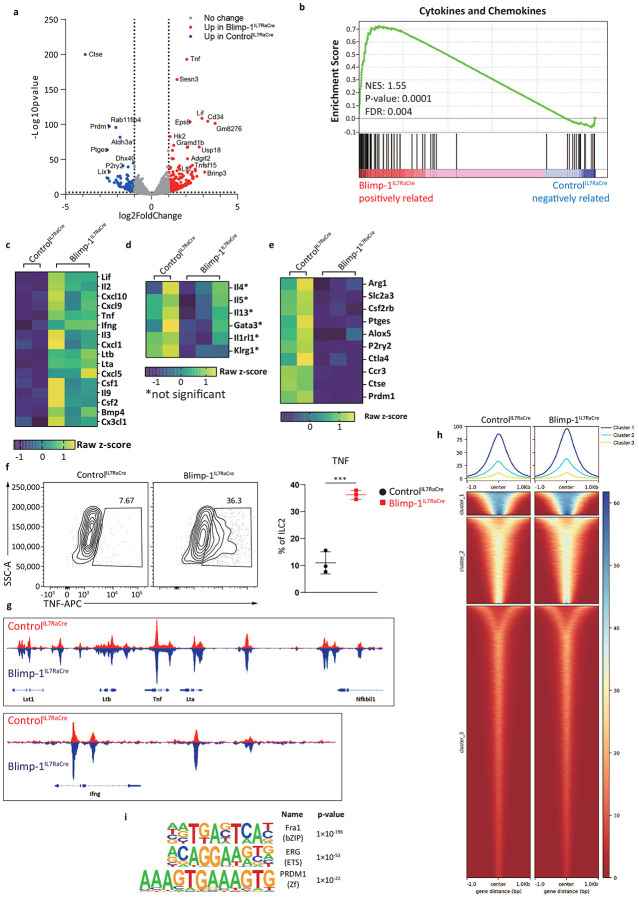
Blimp-1 suppresses Type1 related genes in ILC2s. **a**, Volcano plot from RNAseq comparing Control^IL7RaCre^ and Blimp-1^IL7RaCre^ ILC2s. Threshold was set at log2fold change>1 and log10Pvalue<0.01. **b**, GSEA plot with enrichment in cytokines and chemokines in Blimp-1^IL7RaCre^ ILC2s. NES: 1.55, P-value: 0.0001, FDR: 0.004. **c**-**e**, Heatmap for genes that were upregulated (**c**), no change (**d**), or downregulated (**e**) in Blimp-1^IL7RaCre^ ILC2s. Z-scores were calculated by TPM. **f**, Percentage and quantification of TNFα+ ILC2s from Control^IL7RaCre^ or Blimp-1^IL7RaCre^ with IL-33&25. (n=3) **g**, Representative ATAC-seq peaks near TNFα locus and IFNγ locus.**h**, Tornado plot for ATAC-seq differential peaks comparing Blimp-1 KO with WT ILC2s. **i**, Top results from HOMER motif analysis enriched in open chromatin regions of Blimp-1 KO ILC2s. Each point represents one individual sorting sample from 2 pooled animals. The data are shown as means ± s.d., and present three or four independent experiments. Unpaired t test was performed for **f**. *P < 0.05, *P < 0.01, ***P < 0.001. The specific P values are as follows: for **f**, P = 0.0006.

Given the significant expression of Blimp-1 in ILC2s under several inflammatory conditions, we next aimed to determine the function of Blimp-1 in ILC2s. We crossed Blimp-1 floxed animals to animals that express Cre driven by the IL7Rα gene (Blimp-1^IL7RaCre^), which will delete Blimp-1 in all ILC subsets as well as T cells, B cells, and NK cells^36^. ILC2 numbers in the lung at steady state were similar between Control^IL7RaCre^ and Blimp-1^IL7RaCre^, indicating Blimp-1 was not required for the development of ILC2s (Extended Data Fig.4a). As Blimp-1 was absent in several cellular compartments in these animals, we opted to test the function of Blimp-1 in vitro. Sorted ILC2s from the lungs of Control^IL7RaCre^ and Blimp-1^IL7RaCre^ were activated in vitro for 3 days with IL-33 and IL-25 followed by RNA-seq and ATAC-seq. Differentially expressed genes were defined as log2 fold change >2 and p<0.01. 369 genes were differentially upregulated in Blimp-1-deficient ILC2s, while only 89 genes were downregulated (Fig. 5a, Extended Data Fig.4b). Gene set enrichment analysis (GSEA) showed the top pathway enriched in Blimp-1-deficient ILC2s was in cytokines and chemokines (Fig. 5b). Closer inspection revealed several type 1 associated genes were upregulated in Blimp-1-deficient ILC2s, including *Tnf*, *Lif*, *Ifng*, *Cxcl9*, *Cxcl10*, *Lta*, *Ltb*, *Csf1* and *Csf2* (Fig. 5c). Type 2 genes *Il4, Il5, and Il13*, were not significantly different, however *Il9* was significantly upregulated, consistent with prior reports that Blimp-1 suppresses IL-9 in CD4 T cells and suggesting a possible feedback loop where IL-9 induced Blimp-1 and Blimp-1 then negatively regulates IL-9 (Fig. 5c-d). Several genes of interest were also downregulated, including *Arg1*, *Slc2a3* (Glut3), and *Ccr3* (Fig. 5e).

We confirmed an increase in both TNF and IL-9 in Blimp-1-deficient ILC2s when activated in vitro with IL-33+IL-25 at the protein level by flow cytometry and intracellular cytokine staining (Fig. 5f, Extended Data Fig.4c). In contrast IFNγ was not upregulated at the protein level, possibly due to low baseline transcript expression (Extended Data Fig.4d). Similarly, IL-3, GM-CSF and CCL2 were also upregulated in the supernatant upon ILC2 activation in vitro in Blimp-1^IL7RaCre^ cells compared to controls by Luminex assay (Extended Data Fig.4e-g). Interestingly, we found at the protein level that type 2 cytokines were reduced, although RNAseq analysis did not show differences at the transcript level in these genes, suggesting the possibility that increased type 1 genes indirectly regulated type 2 responses (Extended Data Fig.4h-j).

Blimp-1 is a transcriptional repressor known to recruit the histone methyltransferase G9a to promote H3K9me3, a mark of heterochromatin.^37^ Thus, we hypothesized that Blimp-1 may directly bind to upregulated genes identified by RNAseq to mediate repression. Using ATACseq, we compared open chromatin regions in activated ILC2s from Control^IL7RaCre^ and Blimp-1^IL7RaCre^ animals. In line with increases in gene expression, Blimp-1 deficient ILC2s had increased chromatin accessibility at type 1 cytokine genes including the locus that includes *Lta*, *Tnf*, and *Ltb* as well as the *Ifng* locus (Fig. 5g). Genome wide analysis identified increased accessibility at open regions (clusters 1 and 2) but few newly accessible regions, suggesting Blimp-1 limits gene expression of those genes which may already be accessible, rather than alter the epigenetic landscape by opening or closing regions entirely (Fig. 5h). HOMER motif analysis of differentially accessible regions that were more open in Blimp-1 deficient ILC2s identified the Prdm1 consensus sequence as a top hit, confirming Blimp-1 likely binds directly to the genome at type 1 genes to mediate repression (Fig. 5i). Collectively, these data suggest Blimp-1 functions to limit the accessibility and expression of type 1 genes after ILC2 activation, limiting plasticity and protecting the type 2 transcriptional state of ILC2s under inflammatory conditions.

### Blimp-1 in ILC2s promotes type 2 responses and allergen-driven inflammation

**Extended Data Fig. 5:**
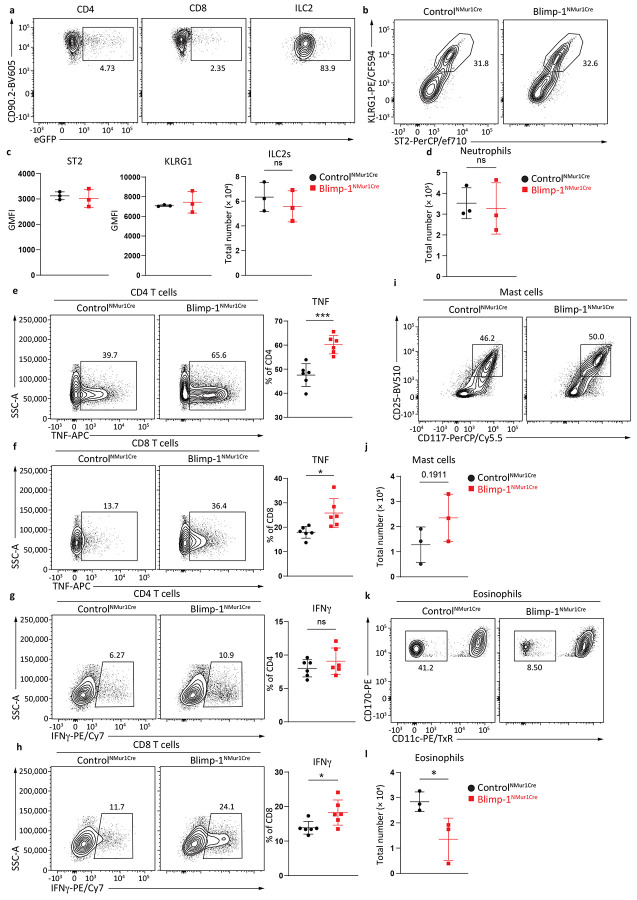
ILC2 Blimp-1 deficient animals exhibit mixed inflammatory responses to papain and tissue alarmins. **a**, Percentage of NMUR1Cre-eGFP+ cells in CD4, CD8 and ILC2s. **b**, Percentage of ILC2s from lung in Control^NMUR1Cre^ or Blimp-1^NMUR1Cre^ animals after IL-33&25. (n=3) **c**, Quantification of ST2 and KLRG1 GMFI in ILC2s, or total ILC2 number from Control^NMUR1Cre^ or Blimp-1^NMUR1Cre^. **d**, Quantification of neutrophils number from Control^NMUR1Cre^ or Blimp-1^NMUR1Cre^. **e**-**h**, Percentage of TNF+ CD4 (**e**), TNF+ CD8 (**f**), IFNγ+ CD4 (**g**) and IFNγ+ CD8 (**h**) from Control^NMUR1Cre^ or Blimp-1^NMUR1Cre^. (n=6) **i** and **k**, Percentage of mast cells (**i**) or eosinophils (**k**) from Control^NMUR1Cre^ or Blimp-1^NMUR1Cre^ animals after papain. **j** and **l**, Quantification of mast cell number (**j**) or eosinophil number (**l**). Each point represents one individual animals. The data are shown as means ± s.d., and present two or three independent experiments. A multiple unpaired t-test was performed for **c**, **d**, **e**, **f**, **g**, **h**, **j** and **l**. *P < 0.05, *P < 0.01, ***P < 0.001, ****P < 0.0001. The specific P values are as follows: for **c**, comparison for ST2: P = 0.6754, comparison for KLRG1: P = 0.6154, comparison for ILC2 number: P = 0.4955; for **d**, P = 0.7788; for **e**, P = 0.0005; for **f**, P = 0.0131; for **g**, P = 0.2968; for **h**, P = 0.0249; for **j**, P = 0.1911; for **l**, P = 0.0494.

**Fig. 6:**
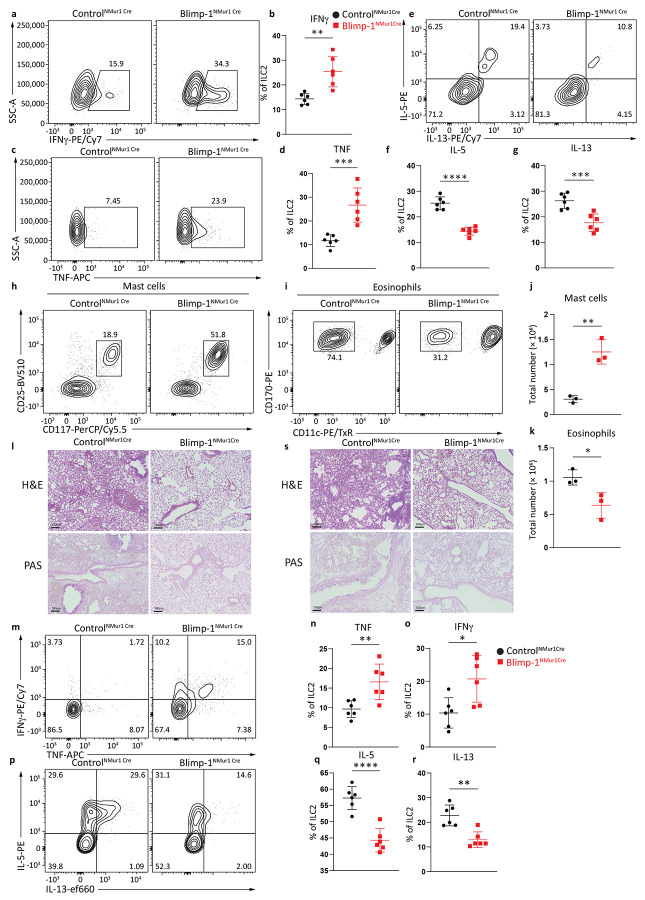
Blimp-1 knockout animals produce more Type1 cytokines upon activation and suppress tumor seeding. **a** and **c**, Percentage of IFNγ+ (**a**) or TNF+ (**c**) ILC2s in Control^NMUR1Cre^ or Blimp-1^NMUR1Cre^ animals after IL-33&25 i.n.. (n=6) **b** and **d**, Quantification of IFNγ (**b**) or TNF (**d**) expression from (**a**) and (**c**). **e**, Percentage of IL-5+ and IL-13+ ILC2s in Control^NMUR1Cre^ or Blimp-1^NMUR1Cre^ animals after IL-33&25 i.n.. **f**-**g**, Quantification of IL-5 (**f**) or IL-13 (**g**) expression from (**e**). **h**-**i**, Representative flow plot for mast cells (**h**) or eosinophils (**i**) in Control^NMUR1Cre^ or Blimp-1^NMUR1Cre^animals. **j**-**k**, Quantification of mast cell number (**j**) or eosinophil number (**k**) from (**h** and **i**). **l**, Representative H&E and PAS staining of Control^NMUR1Cre^ or Blimp-1^NMUR1Cre^ animals after IL-33&25 i.n. **m**, Percentage of IFNγ+ and TNF+ ILC2s in Control^NMUR1Cre^ or Blimp-1^NMUR1Cre^ animals after papain i.n.. (n=6) **n**-**o**, Quantification of IFNγ (**n**) or TNF (**o**) expression from (**m**). **p**, Percentage of IL-5+ and IL-13+ ILC2s in Control^NMUR1Cre^ or Blimp-1^NMUR1Cre^ animals after papain i.n. **q**-**r**, Quantification of IL-5 (**q**) or IL-13 (**r**) expression from (**p**). **s**, Representative H&E and PAS staining of Control^NMUR1Cre^ or Blimp-1^NMUR1Cre^ animals after papain i.n..Each point represents one individual sample. The data are shown as means ± s.d., and present two independent experiments. A two-tailed unpaired t test was performed for **b**, **d**, **f**, **g**, **j**, **k**, **n**, **o**, **q** and **r**. *P < 0.05, *P < 0.01, ***P < 0.001, ****P < 0.0001. The specific P values are as follows for **b**: P = 0.0021; for **d**: P = 0.0007; for **f**, P < 0.0001; for **g**, P = 0.0009; for **j**: P = 0.0029; for **k**, P = 0.0326; for **n**, P = 0.0068; for **o**, P = 0.0133; for **q**, P < 0.0001; for **r**, P = 0.0011.

To extend our analysis of Blimp-1’s function in vivo, we generated animals where Blimp-1 loss was restricted to the ILC2 subset using the Nmur1-Cre strain^38^. The NMur1-Cre locus also expresses eGFP as a marker of NMur1 expression. In the lung, we confirmed that NMur1 is expressed predominately in ILC2s within the ILC compartment and is limited to a small population of activated T cells (less than 5%) (Extended Data Fig.5a). To test the function of Blimp-1 in ILC2s in vivo, we first administered i.n. IL-33 and IL-25 for three days. Expression of ILC2 activation markers including ST2 and KLRG1, nor the total ILC2 number were affected by Blimp-1 loss (Extended Data Fig.5b-c). However, Blimp-1 deficient ILC2s in the lungs produced significantly more IFNγ and TNF in line with the RNAseq results in vitro (Fig. 6a-d). Similar to in vitro protein analysis, Blimp-1 loss led to a significant decrease in the type 2 cytokines IL-5 and IL-13 (Fig. 6e-g). To assess the impact of this shift from type 2 to type 1 cytokines on the recruitment of immune cells to the lung, we investigated cells in the bronchoalveolar lavage. Indeed, there was a shift in the recruitment of mast cells over eosinophils, possibly due to the increases in both IL-3 and IL-9 which are known to recruit and support mast cells as well as decreases in IL-5 (Fig. 6h-k)^39,40^. Neutrophil recruitment was not different between Controls and Blimp-1-deficient ILC2 animals (Extended Data Fig.5d). As early responders to tissue damage, ILC2s influence the inflammatory state and shape T cell responses. Remarkably, we found increased expression of IFNγ and TNF in both CD4 and CD8 T cells in the lung (Extended Data Fig.5e-h). Finally, to assess the impact on lung inflammation, we performed H&E and Periodic Acid-Schiff (PAS) staining to identify mucus in the airways. Blimp-1^NMur1Cre^ animals had reduced mucus in the airways and lymphocytic infiltration into the lung tissue compared to Control ^NMur1Cre^ animals (Fig. 6l).

To extend these findings to a model of allergic inflammation, we immunized Blimp-1^NMur1Cre^ and Control^NMur1Cre^ animals with i.n. papain for three days. Similar to the results with IL-33 and IL-25, we observed increases in IFNγ and TNF and concomitant decreases in Type 2 cytokines IL-5 and IL-13 (Fig. 6m-r). Again, these changes led to a significant decrease in eosinophil recruitment, though mast cell increases were less pronounced in this model (Extended Data Fig.5i-l). However, histology confirmed that Blimp-1^NMur1Cre^ animals had less lymphocytic infiltration and mucus in the airways, and thus less inflammation overall (Fig. 6s). Collectively, these data strongly support a critical role for Blimp-1 in ILC2s to promote allergic responses by maintaining type 2 cytokine production and directly repressing type 1 genes.

## Discussion:

Blimp-1 is a transcriptional repressor with cell type specific functions expressed in immune cell populations such as plasma cells and effector T cells that acts to repress gene programs associated with alternate or prior states^18,37,41,42^. We previously identified a role for Blimp-1 in the earliest stages of Th2 differentiation in response to inhaled allergens^13,43^. Here, we extend the role of Blimp-1 in supporting type 2 responses to allergens to the ILC2 compartment.

ILCs are helper like innate lymphoid cells that lack TCRs and thus antigen specificity but share the transcription factor and cytokine programs identified in CD4 T cells^44^. Accordingly, ILC2s have similar transcriptional profiles with Th2 cells^45^. ILC2s respond to tissue alarmins which rapidly drives the type 2 cytokines IL-5, IL-13 and IL-9 and to some extent IL-4^46^. Lung ILC2s are found in the adventitial spaces adjacent to the airways, spatially placing them where they can readily detect tissue damage signals in response to allergens and recruit inflammatory cells to the airways^47^. ILC2s also locally influence allergen-specific T cells activated in the draining mediastinal LN and recruited to the lung^48^. Together, Th2 cells and ILC2s then recruit inflammatory cells such as eosinophils and mast cells that drive lung inflammation and pathophysiological symptoms of asthma such as goblet cell metaplasia and mucus production, airway restriction and smooth muscle remodeling^49^ .

Our study demonstrated in response to allergens that loss of Blimp-1 in ILC2s led to a shift from type 2 cytokine production to type 1 cytokines. In the acute models of allergic lung inflammation we explored, this led to an overall decrease in inflammation in the lungs. Although we did not find statistically significant decreases in type 2 cytokines by transcript expression in the absence of Blimp-1, both IL-5 and IL-13 were reduced at the protein level. This suggests that Blimp-1 mainly acts to repress type 1 cytokines, and their presence likely indirectly limits type 2 cytokine production. While ILC2 plasticity towards IFNγ producers in settings of viral infection or settings with IL-1β have been described, the mechanism of maintaining type 2 transcriptional fidelity has not been elucidated^6-8^ .

A main feature of ILCs compared to T cells is their ability to rapidly respond and produce cytokines. In vitro, we found Blimp-1 was rapidly upregulated. Acute models in vivo such as papain confirmed Blimp-1 was robustly expressed after a short duration of exposure. In contrast, using a more T cell driven model of allergic asthma, HDM, we found Blimp-1 was upregulated later in the response suggesting chronic stimulation of allergen can additionally support Blimp-1+ ILC2s. Indeed, both ILC2s in the tissue as well as those recruited to the lung from the bone marrow or gastrointestinal tract expressed Blimp-1. Thus, Blimp-1 upregulation is a general feature of ILC2 activation and its timing likely depends on the intensity and timing of tissue alarmins induced by allergen.

In T cells, Blimp-1 is driven by cytokine-Jak/STAT pathways such as IL-12/STAT4 in Th1 cells, and IL-10/STAT3 in Th2 cells^33,34^ . Intriguingly, unlike Th2 cells, we were unable to find a role for IL-10 to induce Blimp-1 (data not shown). Instead, we found IL-9 was necessary and sufficient for Blimp-1 expression in ILC2s. IL-9 is a common gamma chain type 2 cytokine that is known to signal via STAT5 and STAT3^50^ . STAT3 has been identified in several cell types to play a role in promoting allergic lung inflammation in pre-clinical models^51-53^ . Given the role of STAT3 to promote Blimp-1 in Th2 cells downstream of IL-10, it is interesting to speculate that STAT3 likely also plays a key role in Blimp-1 regulation in ILC2s. IL-33 has been shown to drive a non-canonical form of STAT3 activity through serine 727 phosphorylation that leads to mitochondrial localization of STAT3 and impacts on ILC2 metabolism^51,54^ . How IL-9 mediated STAT3 impacts ILC2s is less well understood, though we speculate that Blimp-1 expression may be important for the effects of IL-9 on ILC2s.

Clinically, allergic asthma is associated with type 2 responses, however not all patients have high expression of type 2 mediators, and a subset of patients are refractory to corticosteroid treatment and are classified as having severe disease^55-57^ . Severe asthma patients often do not have high type 2 inflammation but rather exhibit a mixed inflammatory response with increases in IFNγ and other type 1 inflammatory features. A subset of severe asthma patients has increased Th17 cells as well as neutrophils^58^ . Furthermore, mast cells are well known to play a key role in driving inflammation in allergic asthma^59^ . Here, we show loss of Blimp-1 leads to an increase in type 1 cytokines with a concomitant decrease in type 2 cytokines, reminiscent of mixed inflammation observed in some patients with severe asthma. Given the known inflammatory impact of mast cells, it was somewhat surprising to see reduced inflammation in the lung tissue by histology. We speculate that chronic allergen introduction leading to IgE and subsequent mast cell activation may shift the inflammatory impact of loss of Blimp-1 in ILC2s leading to worse disease upon long term allergen exposure.

In conclusion, our study of Blimp-1 in ILC2s in allergic lung inflammation has uncovered an unexpected and critical role for Blimp-1 to promote and protect the type 2 transcriptional identity of ILC2s by actively repressing the gene expression of type 1 inflammatory genes. Future studies using this model to understand the role of mixed inflammatory responses on the lung may be of interest as a model for severe asthma and to understand the contribution of mast cells versus eosinophils on the pathophysiology of the lung in allergic lung inflammation.

## Methods

### Mice

C57BL/6J (000664), B6.Cg-Tg(Prdm1-EYFP)1Mnz/J (008828), B6.129-Prdm1 ^tm1Clme^/J (008100), Il1rl1^tm1Anjm^, Il7r^tm1.1^(icre)Hrr, animals were either purchased from Jackson Laboratories or maintained separately. Il7r^tm1.1^(icre)Hrr, animals were obtained from Hans-Reimer Rodewald (DKFZ). Il1rl1^tm1Anjm^ were obtained from Heth Turnquist (U.Pittsburgh). C57BL/6-Tg(Nmur1-iCre,-eGFP)1Dart/J (038197) animals were obtained from David Artis (Weill Cornell University), now available from Jackson Laboratories. Blimp-1^NMUR1Cre^ animals were generated by crossing with B6.129-Prdm1^tm1Clme^/J (008100). Animals were housed in specific pathogen-free enclosures at the John G. Rangos Sr. Research Center of UPMC Children’s Hospital of Pittsburgh or The Assembly Building under conditions:12 h light–dark cycle, temperature of 19–23°C and humidity of 40–60%. All experiments were approved by the University of Pittsburgh Animal Care Committee and the American Veterinary Medical Association. All animals were over 6 weeks of age, and either matched male or female animals for each experiment. All in vivo experiments were performed independently at least two to three times, with at least three animals per group for one independent experiment. Randomization and blinding were not used. The sample size was determined by power analysis and prior experience. No datapoints were excluded from the analyses.

### Airway inflammation models

A total of 25 μg of lipopolysaccharide-low HDM (Stallergenes Greer) in PBS was given i.n. to animals anesthetized with isoflurane daily for up to 10 days (priming). To induce allergic lung inflammation, animals were immunized i.n. with HDM for 10 days and rechallenged two times for 2–3 days. For papain, 25μg or 40μg of papain (Sigma Aldrich) was used daily for 3 days. For cytokine intranasal stimulation, recombinant IL-33 (Biolegend) and IL-25 (Biolegend) or IL-9 (PeproTech) were used. 0.5μg of each cytokine was diluted in 25μL of sterile PBS and given intranasally to isoflurane-anesthetized animals daily for 3 days. For FTY720 (Enzo life science, BML-SL233-0025), the animals treated with HDM were given 25 μg of FTY720 every other day for 10 days (intraperitoneal, i.p.). Lungs were harvested for evaluation one day after treatment.

### Skin model

For the IL-33&25 mediated model, for sensitization, 0.5μg of recombinant IL-33 (Biolegend) and IL-25 (Biolegend) in 100μL of sterile PBS was subcutaneously injected (s.c.) to shaved flank skin of animals every day for 3 days. Control animals received 100μL of sterile PBS instead of cytokines. Draining lymph nodes were isolated on day 4 for evaluation.

### Small intestinal lamina propria model

For IL-33&25 driven gut ILC2 activation model, 0.5μg of recombinant IL-33 (Biolegend) and IL-25 (Biolegend) in 100μL of sterile PBS was intraperitoneal injected (i.p.) to animals every day for 3 days. Control animals received 100μL of sterile PBS instead of cytokines. Mesenteric lymph nodes (mLNs) were isolated on day 4 for evaluation.

### Inflammatory ILC2s (iILC2s) induction

To induce iILC2s in the lung, 0.5μg of recombinant IL-25 (Biolegend) in 100μL of sterile PBS was intraperitoneal injected (i.p.) to animals every day for 3 days. Control animals received 100μL of sterile PBS instead of cytokines. Lungs were harvested for evaluation on day 4.

### Tissue processing

#### Lung

Animals were anesthetized and sacrificed with intraperitoneal injection of ketamine/xylazine. To collect bronchoalveolar lavage fluid, a catheter was inserted in the trachea, and 0.9ml of PBS was instilled and retracted. BAL was later centrifuged at 2000rpm at 4°C for 10min, the cell pellet was resuspended with 200μL PBS and counted with automated cell counters (Nexcelom Bioscience LLC). Around 4×10^5^ BAL cells were stained for flow cytometry. The lung was perfused with PBS from right ventricle through pulmonary artery. In some experiments, the left lung was tied and removed for flow cytometry. The right lung was inflated and submerged with Safefix II (Thermo Fisher Scientific) for histology or directly digested for flow cytometry. The whole lung was taken down and processed for sorting experiments. For lung cells, a whole lung was digested with 5ml of 0.7mg/mL collagenase A (Sigma-Aldrich) and 0.03mg/mL DNAse I (Sigma-Aldrich) RPMI solution while gently rotating for 45min. And the tissue was processed with gentleMACS dissociator. 2% FBS 2mM ETDA PBS solution was used to stop digestion. RBCs were lysed with ACK lysing buffer (Thermo Fisher Scientific). Cells were filtered through a 70μm filter (CELLTREAT).

#### Small intestinal lamina propria

To harvest siLP ILC2s, the small intestine was isolated, the associated mesenteric tissue removed, and the contents emptied. The tissue was opened with scissors, and Peyer’s Patches were removed for further digestion. Small intestine tissue was rinsed with PBS and then cut into small pieces. The tissues were immersed in media containing 3%FBS, 5mM EDTA, and 0.145mg/mL DTT and incubated at 37°C while stirring at 800rpm for 20min. After the incubation, the tissue and media were filtered, collected and shaken with buffer (2mM EDTA in RPMI1640) for 30sec, then strained again and repeated 3 times. The tissue was collected and digested in digestion buffer with 0.5mg/mL DNase I (Sigma-Aldrich) and 0.1mg/ml Liberase TL (Roche) at 37°C for 25min while stirring. 3% FBS media was used to stop the digestion, and the single-cell suspension was collected through a 70 μm filter. For in vivo gut ILC2 activation experiments, mLNs were isolated and processed for flow cytometry.

#### Skin

To isolate cells from flank skin, animals were shaved and the desired sample was cut and spread with the inner side up on a 10 cm cell culture plate. The blunt end of the forceps was used to remove the fat and cut the tissue into small pieces. Tissue was digested with digestion buffer (collagenase XI (2.5mg/mL) (Sigma-Aldrich), hyaluronidase (0.25mg/mL) (Sigma-Aldrich) and DNase I (0.05mg/ml) (Sigma-Aldrich) in RPMI 1640) at 37°C while shaking at 300rpm for 1h. After digestion, a gentleMACS dissociator was used to dissociate cells to a single-cell suspension and filtered through a 70μm filter. For in vivo skin ILC2 activation experiments, draining LNs were taken down and processed for flow cytometry.

#### Spleen and lymph node

Spleen and LNs were isolated in RPMI-1640 (BioWhittaker) and crushed through 40μm filters. For spleen, RBCs were lysed with ACK lysing buffer (Thermo Fisher Scientific).

### Flow cytometry

Single-cell suspensions were collected as above. Cell number was determined by an automated cell counter (Nexcelom Bioscience LLC) and resuspended to 1×10^7^cell/ml and plated into 96-well plates for staining. Cells were washed with PBS (BioWhittaker) twice before staining. Cell pellets were resuspended with 40μL HBSS containing 0.5% Fc Block and antibody cocktail for 30 min at 4 °C. For intracellular staining, cells were stimulated with 50ng/ml PMA, 1 μg/ml ionomycin, brefeldin A (GolgiPlug, BD), and monensin (Ebioscience^™^) at 37°C for 2h. The same concentration of BFA and monensin was used for unstimulated control. Cells were stained with antibodies for surface markers and live/dead, fixed at 4°C with BD Cytofix/Cytoperm (BD Bioscience) and stained for cytokines and transcriptional factors with BD Perm/Wash buffer (BD Bioscience). BD LSRFortessa flow cytometer was used for flow analysis, and data were further analyzed with FlowJo (v10.8.1 and v10.10.0).

### Fluorescence-activated Cell Sorting

Cells were resuspended with 2% Fetal Bovine Serum (FBS), and 2 mM EDTA PBS buffer (EasySep buffer), and lineage negative cells were enriched using EasySep Mouse Hematopoietic Progenitor Cell Isolation Kit (Stemcell) with recommended protocol. Cells were then stained for surface markers and ILC2s (live, lineage-, CD90.2+, CD3-) were sorted on a Sony MA900 Cell Sorter. Purity was checked by a double sorting.

### In vitro ILC2 culture and stimulation

ILC2 cells were isolated by FACS from naive or treated mouse lungs as described above. 15,000 cells were plated per well in 96-well plate and cultured with cRPMI (RPMI-1640 containing 10%FBS 1% penicillin/streptomycin 2mM L-glut 50mM HEPES) plus 20ng/mL recombinant mouse IL-2. Additional cytokines were added at the same concentration for further stimulation. Cells were collected after indicated days of culture for analysis by flow cytometry.

### RNA sequencing and analysis

For bulk RNA sequencing, ILC2s were flow sorted ex vivo from lung and 15,000 ILC2s from each sample were cultured in complete RPMI with or without IL-33 for 24 hours or IL-33+IL-25+IL-2 for 72 hours. RNA was isolated with RNeasy Plus Micro Kit (74034, Qiagen). cDNA was prepared using the SMART-seq v4 Ultra Low Input RNA Kit for Sequencing, (Clontech Laboratories). Sequencing libraries were prepared using the Nextera XT DNA Library Preparation kit (Illumina). Cluster generation and 75-bp paired-end, dual-indexed sequencing was performed on an Illumina NextSeq 2000. FastQC was used to perform a quality assessment on all fastq files. The mouse reference genome (GRCm38) was downloaded from Ensembl. Adaptors were trimmed using cutadapt v1.18 and reads were aligned using HISAT2 v2.1.0. Raw count values were generated using Subread, and gene expression values were normalized using transcripts per million. DESeq2 was applied to raw counts transcriptome data in a pairwise manner to determine differentially expressed genes. These gene lists were compiled to make a master list of all differentially expressed genes. Log_2_ transformed normalized counts were then displayed as a heatmap to display expression of differentially expressed genes. Metascape^60^ and Gene set enrichment analysis (GSEA)^61^ were used to identify pathways associated with gene sets.

### ATAC sequencing and analysis

For ATAC sequencing, ILC2s were flow sorted and 15,000 cells cultured in complete RPMI with IL-33+IL-25+IL-2 for 72 hours. ILC2s were collected for OMNI ATACseq protocol^62^ . Sequencing libraries were prepared using the Nextera XT DNA Library Preparation kit (Illumina). Cluster generation and 101-bp paired-end, dual-indexed sequencing was performed on an Illumina NextSeq2000. FastQC was used to perform a quality assessment on all fastq files. The mouse reference genome (GRCm38) was downloaded from Ensembl. Adaptors were trimmed using cutadapt v1.18. Raw count values were generated using Subread, reads were aligned to the reference genome using Bowtie2 v2.3.4.2. Duplicates were removed using Picard, and regions from the ENCODE Blacklist were removed using bedtools intersect. Peaks (narrow) were called using MACS2 v2.1.1 with a p-value cutoff of 0.05. Irreproducible discovery rate (IDR) using a threshold of 0.05 to identify reproducible peaks. IDR peak files of all samples were merged to create a master list of peaks which together with alignment files were used to generate tag counts for each replicate (bedtools coverage). DESeq2 was applied to raw tag count data for called peaks in a pairwise manner to determine differential peaks. These peak lists were compiled to make a master list of all differential peaks for each mark. Peaks were annotated to the nearest gene using ChIPpeakAnn^63^ . Heatmaps were generated using deepTools2 (v.3.3.0)^64^. Bam files were combined across sample replicates. Samples were normalized by bins per million (BPM) and converted to bigwig files. Heatmaps were peak centered in a 2,000 bp region by computing matrix from normalized and merged bigwig files for all peaks of each mark. (computeMatrix reference point –referencePoint center -a 1000 -b 1000) and visualized (plotHeatmap). Genome browser tracks are visualized from bigwig files using integrative genomics viewer^65^.

### Luminex assay

ILC2s were sorted as above and stimulated with IL-33 and IL-25 for 3 days. Cells were stimulated with 50ng/ml PMA and 1 μg/ml ionomycin for 2 hours and supernatants collected. Bio-Plex Pro Mouse Cytokine 23-plex Assay Kit (#M60009RDPD, Bio-Rad) was performed according to the manufacturers protocol, and data collected by Bio-Plex^®^ MAGPIX^™^ Multiplex Reader.

### Histology

Lung tissue was fixed with Safefix II (Fisher Scientific) and embedded with paraffin. Hematoxylin and eosin (H&E) and periodic acid-Schiff (PAS) were used to stain the slides at Pitt Biospecimen Core at UPMC Shadyside or at the Rangos Histology Core at UPMC Children’s Hospital of Pittsburgh. Photos of slides were taken using an Olympus IX83 microscope.

### Analysis of pSTAT3, pSTAT4, pSTAT5 and pSTAT6 expression

For pSTAT3, pSTAT4, pSTAT5 and pSTAT6 detection in vitro, the cells were fixed using BD Phosflow Lyse/fix buffer (558049), followed by permeabilization with BD Perm buffer III (BD Biosciences, 558050) and stained with phosphoantibodies. Naïve T cells stimulated with cytokines for 30 mins were used as positive controls.

### Antibody blockade

Animals were injected i.p. with 0.2 mg of anti-IL9 mAb (BE0181- 9C1, BioXCell) on days 1 and 3 during IL-33 and IL-25 immunization. The same amount of IgG21 isotype control antibody (BE0083-MOPC-21, BioXCell) was injected i.p. to control animals.

### In vitro JAK inhibitor treatment

Sorted ILC2s from Blimp-1 YFP animals were cultured in cRPMI with 20ng/mL recombinant mouse IL-33 with or without Ruxolitinib (5μM) (HY-50856, medchemexpress).

### Cell mixing and transwell co-culture

Sorted ILC2s from Il1rl1^tm1Anjm^ animals were mixed with ILC2s from control animals in a 1:1 ratio and plated in 96-well plate. ILC2s were treated with 20ng/mL recombinant mouse IL-33 in cRPMI for 3 days. Cells were collected from the plate for flow analysis on day 4. ST2 surface staining was used to distinguish the genetic source of cells. For transwell co-culture, a 96-well with 0.4μm polycarbonate (PC) membrane inserts (3391, Corning) were used. Sorted ILC2s from Il1rl1^tm1Anjm^ animals were plated in bottom wells (receiver plate) and equal number of sorted control ILC2s were plated in upper wells (insert plate). ILC2s were treated with 20ng/mL recombinant mouse IL-33 in cRPMI for 3 days. Cells were collected for flow analysis on day 4.

## Extended Data

## Data Availability

All RNAseq and ATACseq described in the paper are available at NCBI GEO RNAseq [GSE291231, Reviewer token: ehwvumawljmvpyl] ATACseq [GSE291230, Reviewer token: cjcrycyebhkzrij].
